# Defining the Components of the Deltoid Ligament (DL): A Cadaveric Study

**DOI:** 10.7759/cureus.23051

**Published:** 2022-03-10

**Authors:** Essam E Ismail, Radi A Al Saffar, Kamaluddin Motawei, Sanket D Hiware, Khwaja Moizuddin, Siraj A Shaikh, Sujata B Bayer, Yasser Alharbi, Rashid A Aldahhan, Syed R Daimi

**Affiliations:** 1 Anatomy, Imam Abdulrahman Bin Faisal University, Dammam, SAU; 2 Anatomy, Imam Abdulrahman Bin Faisal University, Dammam , SAU; 3 Basic Medical Sciences, College of Medicine-Jeddah, King Saud Bin Abdulaziz University for Health Sciences College of Medicine, Jeddah, SAU

**Keywords:** medial ankle ligaments, anatomy, intermediate tibiotalar ligament and deep posterior tibiotalar ligament, anterior tibiotalar ligament, superficial posterior tibiotalar ligament, tibiotalocalcaneal ligament, deltoid ligament

## Abstract

Background: The deltoid ligament (DL) is a strong triangle-shaped ligament with a complex fascicular arrangement. Understanding the morphological and/or functional typing of the DL structure is hindered by a paucity of clear, quantitative, and reproducible data and is further complicated by inconsistent terminology use. The aim of this work was to describe different components of the DL using strict identification criteria.

Methods: Thirty embalmed cadaveric ankles of both sides were dissected on all sides and studied by using gross examination, micro-dissection, and light microscopy by tracing the fascicular pattern of each under 6X magnification.

Results: Six ligamentous bands were identified. The tibiotalocalcaneal ligament (TTC) and the superficial posterior tibiotalar ligament (sPTT) were two superficial variants and the anterior tibiotalar ligament (ATT), the anterior tibiotalonavicular ligament (ATTN), the intermediate tibiotalar ligament (ITT), and the deep posterior tibiotalar ligament (dPTT) were four deep variants. The TTC was identified in all 30 embalmed cadaveric specimens. Five additional ligamentous bands (ITT, sPTT, dPTT, ATT, and ATTN) were variable findings in the current cohort.

Conclusion: This study presents six ligamentous bands as a regular finding and five additional ligamentous bands as variable findings in the dissected specimen. This data could assist in the radiological diagnosis of DL injuries and advanced procedures related to its surgical repair and reconstruction.

## Introduction

The deltoid ligament (DL) is a strong, broad ligament with a complex fascicular arrangement. It spans from the medial malleolus to the calcaneus, navicular, and talus bones, thereby creating a triangular shape. Previous qualitative and quantitative observations have demonstrated that this ligament is a significant stabilizer of both the medial tibiotalar joint and the entire tibiotalocalcaneal joint complex [1,2].

The proximity of the DL to the fibrous sheaths surrounding the tibialis posterior and ﬂexor digitorum tendons creates problems in differentiating DL components from its surroundings [2,3]. Due to the difficulty in isolating the bundles, the interpretations of the anatomy of this ligament are numerous and controversial.

The commonly accepted view is that the DL consists of two layers: a superficial layer and a deep layer. Functionally, the superficial layer restricts the propensity of the talus to move into a valgus position, resisting eversion of the hind foot, whereas the deep layer is the principal restraint of the talus against external rotation [4,5].

Recently, Yammine [5] and Won et al. [6] described the DL using the ligament attachment sites as a guide to identify and illustrate the individual components. These include the tibio-calcaneo-navicular ligament (TCN; a unification of the traditional tibionavicular, tibiospring, and tibiocalcaneal ligaments), the superficial posterior tibiotalar ligament (sPTT), the deep posterior tibiotalar ligament (dPTT), and the deep anterior tibiotalar ligament (dATT). This classification has been deemed more functional and reproducible than the traditional classification [5,6].

The tibionavicular ligament (TNL) represents the most anterior part of the DL. It arises from the ventral border of the anterior colliculus and ends onto the dorsomedial side of the navicular bone. Accordingly, the TNL has been divided into an anterior superficial tibiotalar fascicle, and a tibionavicular fascicle. The former fascicle attaches to the talus. Consequently, considering the anterior part of the superficial layer of the TNL as the TNL may be less perplexing while bearing in mind that it could possibly be attached to the talus [6].

The aim of the current study is establishing the standardized description for the consistently and variably occurring ligamentous bands of DL by cadaveric dissection method.

## Materials and methods

Thirty traditionally embalmed cadaveric ankle specimens were dissected at the Anatomy Research Laboratory, Imam Abdulrahman Bin Faisal University, Dammam, Saudi Arabia. In total, 18 specimens were right ankle and 12 were left ankle and included 6 pairs. The donors’ mean age at death was 75.87 ± 10.22 years (59-91 years). Sixteen specimens were taken from male cadavers and 14 specimens were taken from female cadavers. The anatomical disposition of the DL was considered by gross observation and light microscope. 

The medial aspect of the ankle joint was dissected using standard surgical tools under good lighting to maintain perfect visualization. The skin, muscles, and extensor retinaculum were removed to expose the capsule of ankle joint. Under 6X magnification (OPTIKA, ST-50led, Italy), individual ligamentous fascicles were traced from its origin to insertion, and any irregular (non-fascicular) tissue was removed. The periosteum was also removed for clear recognition of the actual ligamentous attachment sites. A tripod-mounted digital camera (Canon 550D, Japan) was used to shoot all the specimens.

Regular expressive terms were applied to name each ligamentous band according to the guidelines of the Federative Committee on Anatomical Terminology [7]. The names were also modified by the research group based upon the overall appearance of the ligamentous fascicles where they crossed over the tarsal bones. Therefore, each name reflects the potential attachment of a ligament to a bony landmark. Data was collected and processed by using IBM Statistical Package for the Social Sciences (SPSS) version 23. Descriptive data are presented as frequencies (percentages) for discrete variables and as means (standard deviation (SD)) for continuous variables.

## Results

Over the medial side of the ankle, the DL was identified as a complex of variable ligamentous attachments. Six ligamentous entities were collectively isolated within the entire collection of samples comprising the DL. These entities included two superficial variants (the tibiotalocalcaneal ligament (TTC) and the sPTT), and four deep variants (the anterior tibiotalar ligament (ATT), the anterior tibiotalonavicular ligament (ATTN), the intermediate tibiotalar ligament (ITT), and the dPTT) (Table 1).

**Table 1:** Various ligamentous composition of the deltoid ligament (DL) complex.

Ligament Variant	n (%)
The tibiotalocalcaneal ligament (TTC)	30 (100)
The anterior tibiotalar ligament (ATT)	12 (40)
The anterior tibiotalonavicular ligament (ATTN)	18 (60)
The intermediate tibiotalar ligament (ITT)	9 (30)
The superficial posterior tibiotalar ligament (sPTT)	27 (90)
The deep posterior tibiotalar ligament (dPTT)	27 (90)

The nomenclature of different fascicles of DL were based upon the ligamentous orientation and attachment sites provided by the previous studies. In certain samples, this nomenclature has been modified by the research group to describe the extension of some fibers to new attachment as in ATTN, which considered the novelty of this article.

The TTC

**Figure 1:**
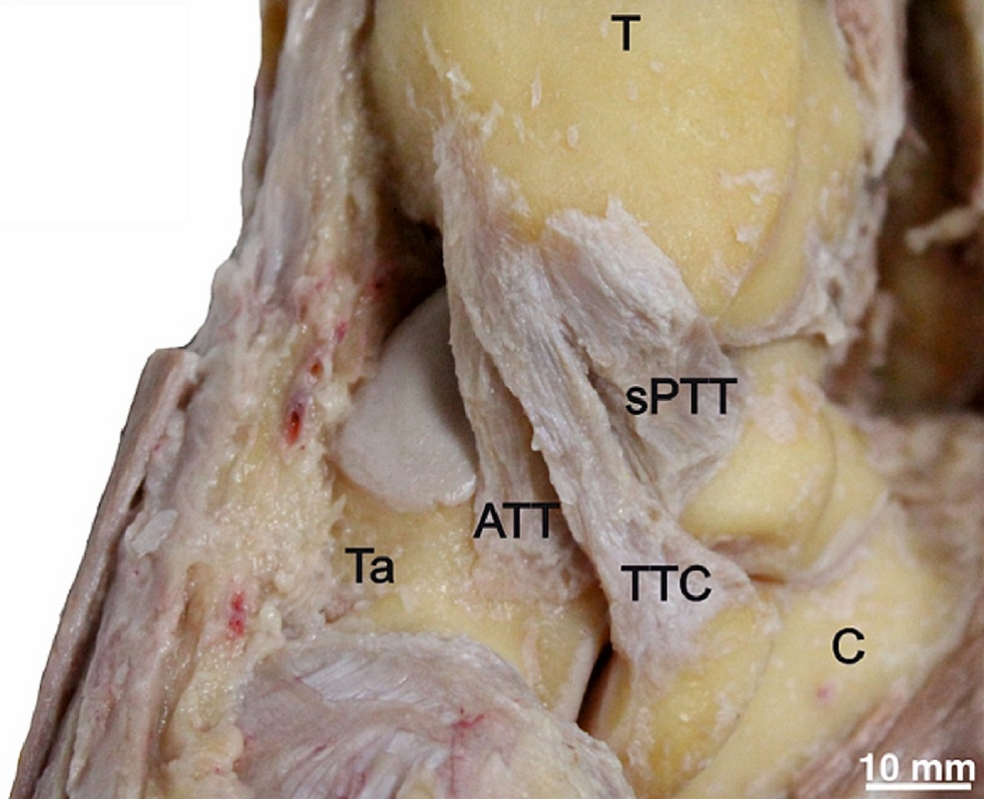
The tibiotalocalcaneal ligament (TTC), superficial posterior tibiotalar ligament (sPTT), and anterior tibiotalar ligament (ATT). T = tibia, Ta = talus, C = calcaneus

**Figure 2:**
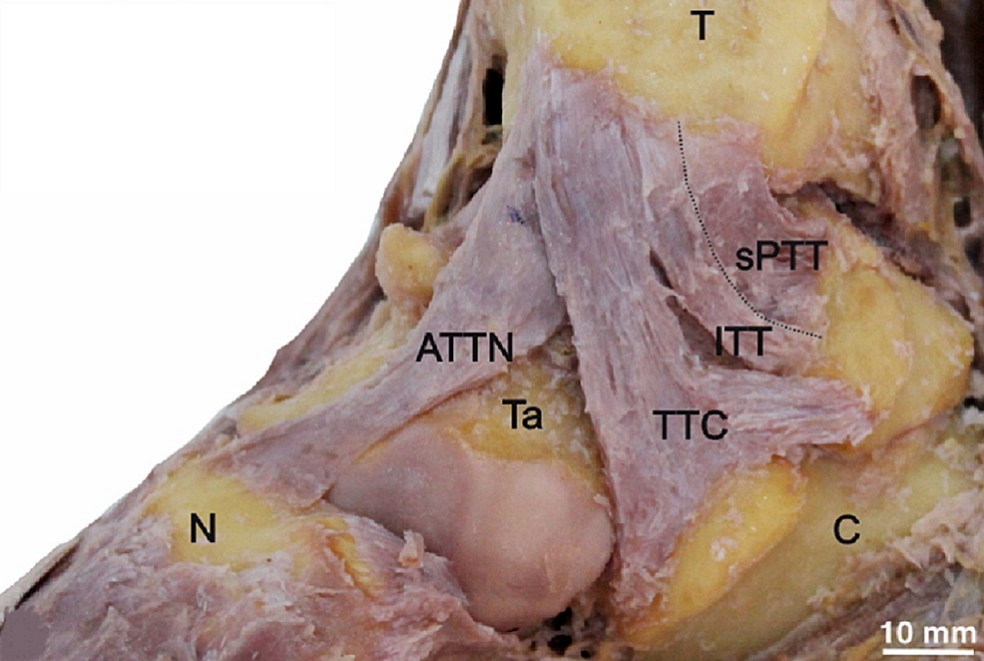
The anterior tibiotalar ligament with navicular extension (ATTN) is overlapped by TTC. The intermediate tibiotalar ligament (ITT) intervenes between the TTC and the superficial posterior tibiotalar ligament (sPTT). T = tibia, Ta = talus, C = calcaneus and N= navicular.

**Figure 3:**
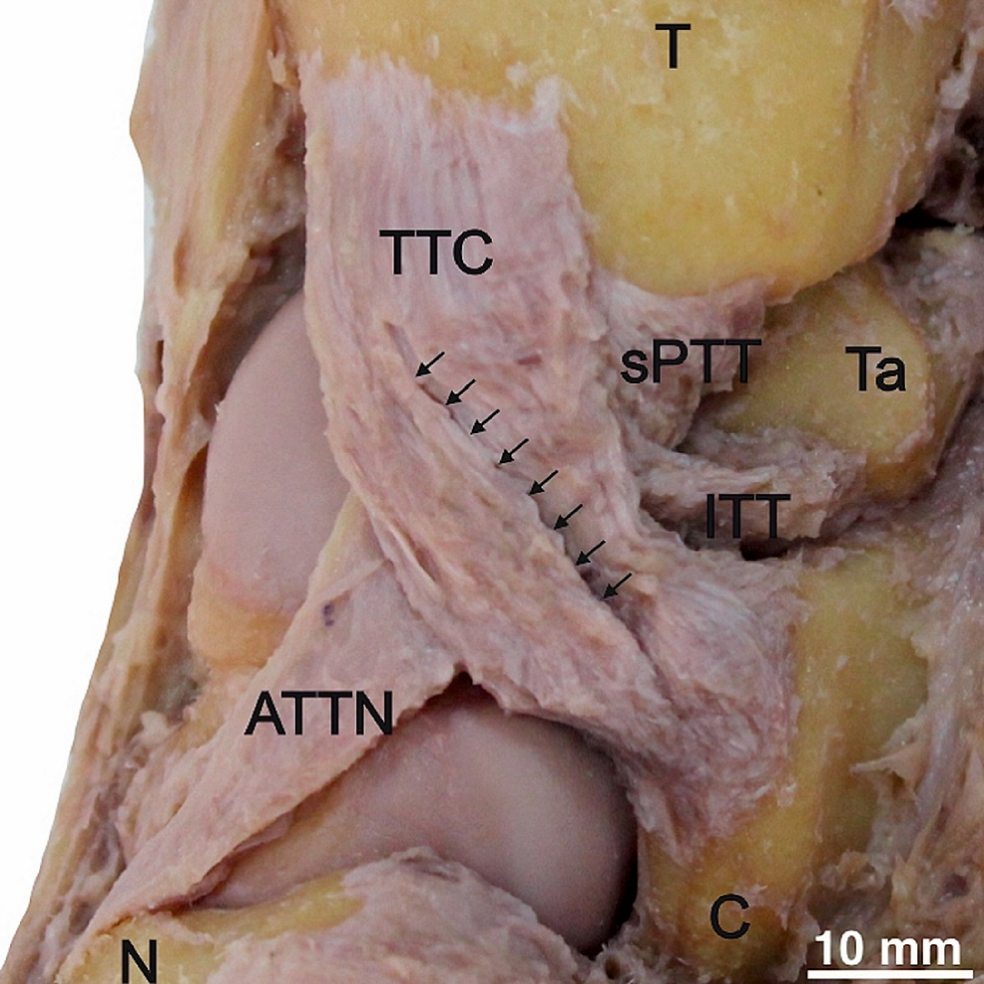
Sulcus (arrows) between two bands of TTC. The anterior tibiotalar ligament with navicular extension (ATTN), superficial posterior tibiotalar ligament (sPTT), and the intermediate tibiotalar ligament (ITT). T = tibia, Ta = talus, C = calcaneus and N = navicular.

The TTC was consistently identified in all the samples (n=30, 100%). It showed a proximal attachment over the anteromedial side of the medial malleolus and extended like a sling (pulley) over the talus and was distally attached to the superiomedial aspect of the sustentaculum tali (Figure 1). However, a TTC with more complex talar attachments was also identified (n=6, 20%). In that TTC, the most posterior ligamentous fascicles extended toward the medial edge of the sulcus tali (Figure 2). A double banded TTC was also isolated (n=6; 20%). The two bands extended parallel to each other and were separated by a non-fascicular tissue (Figure 3). 

 

The ATT

The ATT (n=12, 40%) was recognized with a proximal attachment over the distal anterolateral margin of the medial malleolus. The ligament extended distally over the anteromedial aspect of the talus, anterior to the sPTT (Figure 1).

The ATTN

The ATTN was recognized in (n=18, 60%), which revealed a similar attachment of ATT but expanded distally to reach the medial side of the anterior surface of the navicular bone (Figures 2, 3). 

The ITT

The ITT (n=9, 30%) was observed lying between the ATT/ATTN and sPTT. It had a more vertical orientation than the other tibiotalar ligaments. Its tibial attachment lay over the anteromedial side of the medial malleolus, medial to the ATT connection and under the TTC fascicles (Figures 2, 3). The talar attachment of the ITT reached the anterior part of the sulcus tali edge. However, it showed a higher affinity for coexistence with the ATTN (n=6, 20%) than the ATT (n=3, 10%).

The sPTT

The sPTT (n=27, 90%) was isolated with a proximal attachment over the posterior aspect of the medial side of the medial malleolus. The ligament extended distally to the anteromedial margin of the posterior process of the talus (Figures 1, 3).

The dPTT

**Figure 4:**
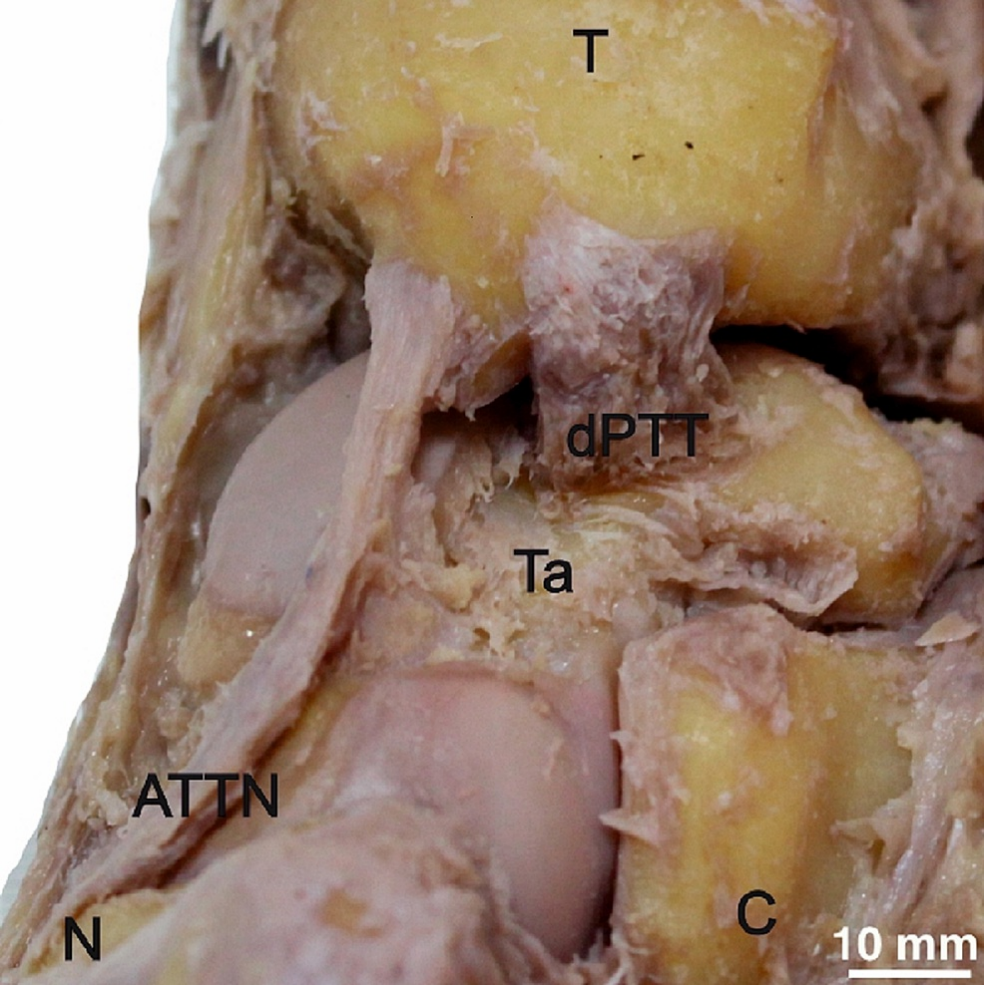
Deep posterior tibitalar ligament (dPTT) and anterior tibiotalar ligament with navicular extension (ATTN). T = tibia, Ta = talus, C = calcaneus, N = navicular.

The dPTT (n=27, 90%) was identified under the sPTT and was separated from it by a fatty layer. It was found proximally attached over an anteromedial side of the medial malleolus, deeper to the sPTT attachment. It extended obliquely to the posterior aspect of the articular facet of the medial malleolus, over the medial edge of the trochlea of the talus (Figure 4). 

 

## Discussion

The current study provides an anatomical description of the DL, including its origins and the insertions of the various bands in relation to the osseous landmarks and spatial relationships. Fibroblast-like cells which are termed as tenocytes or ligament fibroblasts are the main type of cells in the tendon and ligaments. They are located in the parallel chains of the collagen fibrils. These ligament cells have a low rate of proliferation. They are quiescent under normal developmental conditions. The mechanical load affects the extracellular matrix production. Scx, Mkx, and Egrl have been identified as the effective regulators of the development of the ligament and tendons in recent advanced researches on ligament and tendon biology. They have also been identified to affect the related homeostasis. They include types I, II, II, and IV collagen. These collagen fibers are also found to be produced during embryogenesis related to formation of ligament and tendon tissues. The mechanical load at the ankle joint can be variable during the embryological development and hence it can affect the newly found regulators like Scx, Mkx, and Egrl which in turn can affect the variable development of tendons and ligaments in the region of ankle [8].

Since the spring ligament was considered anatomically independent of the DL [9], it was not examined. The dissection of the DL is a complex procedure. A lot of difficulty is encountered when separating it from the joint capsule and tendon sheet of the tibialis posterior and flexor digitorum longus muscles. This explains, at least in part, the varied interpretations made previously for its multiple ligamentous bands [10,11]. Hence, the aim of this cadaveric study was to focus on the anatomical differences of the DL constituent bands.

In the present study, six ligamentous bands composing the DL were collectively isolated within the samples: two superficial and four deep variants that coincided with previous descriptions. However, four components belonging to the superficial layer and two to the deep layer were identified in the previous reports [9,11]. These findings partially align with descriptions in the literature where six different components of the DL were determined, but four components belonged to the superficial layer and two for the deep layer [9,11]. By contrast, eight different bands of the DL have been also described: the tibiocalcaneal ligament, the anterior talotibial ligament, the TNL, the superficial posterior talotibial ligament, the deep posterior talotibial ligament, fibers to spring ligament (plantar calcaneonavicular), and a deep band to the tibiocalcaneal [9,12,13]. In 1979, Pankovich and Shivaram, after dissection of 16 cadaveric ankles, described three bands for the superficial layer (superficial talotibial, calcaneotibial, and naviculotibial ligaments) and two for the deep layer (deep posterior talotibial and deep anterior talotibial ligaments) [14,15].

The prevalence of each band of the DL complex has been reported by a number of authors [9,10,15,16]. In the current work, the superficial and deep layers of DL originated from the anterior and posterior colliculi of the medial malleolus, in agreement with the findings of several authors [17-20]. The TTC was consistently found in all the examined specimens, again in alignment with the findings of other studies [13,16,17]. However, the TTC was named the TCN, as it included the tibiocalcaneal, tibionavicular, and tibiospring ligaments [13]. The current findings showed that the TTC arises from the ventromedial half of the anterior colliculus and terminates into the superomedial aspect of the sustentaculum tali, whereas it has been reported previously to be inserted into the dorsal margin of the sustentaculum tali [16,18-20].

This work confirmed the presence of the ATT in 40% of the examined ankles. This percentage contrasted with the value of 85% recorded by Won et al. [6] and 80% reported by Zamperetti et al. [13], but this may reflect racial differences. The ATT was isolated in 60% of the samples in the current study and showed an attachment to the navicular bone; therefore, it was defined as the ATTN. This attachment has been described in the literature as the tibionavicular band of the DL, with a prevalence of 90% [5].

The ITT was isolated in the present study in 30% of the studied ankles, in accordance with previously published reports [13,19,21,22]. The ITT was isolated from 20% of the samples but was referred to as the deep intermediate tibiotalar ligament (dITT). It was considered to be a secondary bundle of the dPTT and was not always identified in all specimens in previous studies [23-26].

The sPTT was found in 80% of the specimens in the present study, in agreement with previous reports [18,27,28]. Conversely, this band was isolated in 100% of the studied samples in another study [13]. The sPTT was found to begin from the dorsal part of the anterior colliculus of the medial malleolus and to end into the medial tubercle of the dorsal process of the talus [13,29,30]. This description corresponds to that determined in the current study.

The dPTT was observed in only 80% of the samples in this study. This observation differs from those of other studies, where a similar band was reported over the entire sample group (100%) [13,16]. Regarding its attachment, the dPTT was reported by Zamperetti et al. [13] to start from the back of the anterior colliculus or the posterior colliculus. It then terminates into the depression of talus below the articular for the medial malleolus. This was confirmed by the current study.

The absence of precise information on the origins and insertions of the different bands of the DL could have given rise to inaccurate data from secondary repairs or reconstructions of the DL complex compared with lateral ankle ligament procedures. The present study provides details about the bony attachments of the different bands of DL, that could assist in the radiological diagnosis of DL injuries and advanced procedures related to its surgical repair and reconstruction. An MRI analysis, because of its increased accuracy, is a better diagnostic test for diagnosing deep deltoid rupture [31]. This study also offers a detailed approach for different band identification and isolation, using both gross observation and micro-dissection methods. However, the dissection approach is invasive, so it creates a chance of inter-observer variability regardless of how strictly the criteria are followed. The full medical history of the cadavers used in dissection is not known and hence it may affect the findings.

## Conclusions

The DL is the complex fascicular arrangement of various ligaments providing stability to the ankle joint. The present study made an attempt to provide an anatomical description to various ligamentous bands comprising DL in relation to bony landmarks. We found six ligamentous bands forming the DL: two superificial bands (TTC and sPTT) and four deep bands (ATT, ATTN, ITT, and dPTT). The provided data could assist in the radiological diagnosis of DL injuries and advance procedures related to its surgical repair and reconstruction. Further histological investigation is required before firm conclusions can be drawn regarding the ligamentous band composition and their actual attachment sites. Further studies can be conducted using bigger sample size by cadaveric and radiological methods to study the different parts of the DL and its variations. The sexual dimorphism of DL can also be studied by taking paired specimen of considerable sample size.
